# DO THE PRESENCE OF ADULT CHILDREN AND THEIR MARITAL STATUS MATTER FOR ACCESS TO THE PUBLIC LONG-TERM CARE SYSTEM?

**DOI:** 10.1093/geroni/igz038.3422

**Published:** 2019-11-08

**Authors:** Tomoko Wakui, Suguru Okubo, Nanako Tamiya, Taeko Watanabe, Tatsuro Ishizaki, Ichiro Kai

**Affiliations:** 1 Tokyo Metropolitan Institute of Gerontology, Tokyo, Japan; 2 BMS Yokohama, Yokohama, Kanagawa, Japan; 3 Department of Health Services Research, Faculty of Medicine, University of Tsukuba, Tsukuba, Ibaraki, Japan; 4 Department of Health Services Research , Faculty of Medicine, University of Tsukuba, Tsukuba, Ibaraki, Japan; 5 The University of Tokyo, Tokyo, Japan

## Abstract

Knowing how the presence of family affects access to the public long-term care system is important for evaluating the adequacy of the system. This study examined the relationship between the presence of adult children and their marital status, and access to a public system by examining the gap between self-reported care needs and the official certification as needing care under the Japanese public long-term care insurance system. Data from Japan’s 2016 Comprehensive Survey of Living Conditions were used. A total of 23,466 older adult claimants, aged 65 years and older were analyzed. Outcomes were whether or not claimants were officially certified as needing care under the system, and the relationship of the presence of both live-in and live-out children and their marital status were examined controlling for claimants’ age, gender, education, financial status, and physical and cognitive conditions. Females comprised 64.8% of the sample, and the average age was 83 years (SD=7.8). The percentage of claimants living with a single or married child were 25.2% and 26.9%, respectively, and 60.1% were parents of children who lived independently. The percentage who were officially certified as needing care was 68.5%. Logistic regression analysis revealed that claimants with a live-in child were less likely to be officially certified as needing care, and claimants with live-in a single child were less likely to be officially certified compared with those with a married child. Live-in single children may provide long-term care by themselves, and the excess burden on them needs to be further investigated.

